# The Progress of the Tuberculosis Committee

**Published:** 1912-05-04

**Authors:** 


### THE PROGRESS OF THE TUBERCULOSIS COMMITTEE.

The Departmental Committee on Tuberculosis appointed in February has already issued an interim Report which deals with many important aspects of the tuberculosis question, and outlines an ambitious policy for its solution. This Report deserves the careful attention of everyone who desires to keep abreast of the times on this topic of truly national importance; and to medical and institutional workers it is, naturally, of added interest. It is, perhaps, too much to hope that at present the discussion of measures such as those favoured by this Committee should take precedence with the "man in the street" over the sordid squabbles and undignified wrangling engendered by the party system of politics. Such a Utopian state of affairs is still a long way off; but the health age has already dawned, light is steadily displacing darkness in many ways, and in a generation or two the sun of hygienic truth will certainly be fully risen.

The scheme which the Committee unanimously recommends contains two main divisions or parts. One is for the detection of tuberculosis in its earliest stages by means of tuberculosis dispensaries; the other for the provision of sanatoria and other institutions for the treatment of selected cases from the dispensaries. It is suggested that the dispensary shall act as a centre for disseminating general information about tuberculosis as well as a clinic for diagnosing cases and for curative treatment. One dispensary, the Committee opine, will be required for every 150,000 to 200,000 of population in urban centres. In the country, it is evident that the relative sparsity of population would necessitate much more liberal provision than this.

As regards the work of the tuberculosis dispensary, the Committee are, in our opinion, rather too optimistic; but the fault is, after all, on the right side, and their sanguine estimations will be realised, let us hope, in the course of a few years. The capital outlay on a dispensary where an existing building can be adapted for the purpose should not exceed £250, and might, the Committee hope, be less. Special emphasis is laid upon the necessity of obtaining suitably qualified and experienced men for the senior appointments in connection with the dispensaries and sanatoria. We trust that equal emphasis will be laid, when the time comes, on the necessity of paying them adequate salaries. To sweat the medical man is the very falsest of economies, as those who benefit under the Insurance Act will find out at the cost of their health and lives if the Government carries out the threats which have recently been hurled against the medical profession. It is poor tactics to sweat the Insurance Act doctor, so that in order to live he must scamp his work; and then to erect this costly machinery of dispensaries to treat those serious cases whose initial, curable symptoms he had not time to diagnose.

The second portion of the scheme is concerned with the provision of sanatoria, hospitals, and other institutions for tuberculosis; and here the Committee are including surgical and other forms of non-pulmonary tuberculosis. They suggest the provision of one bed in a sanatorium for every 5,000 of population; the average cost is estimated not to exceed £150 per bed, and that of maintenance at twenty-five to thirty shillings a week. A special point is made of the provision of institutions for children. To the administration of these sanatoria the Report devotes detailed attention; and it is suggested that the unit area should generally be a county or a county borough, or groups of them, and that the organisation should be entrusted to the county or county borough councils (or to a joint committee of them in cases of combinations).

While the Committee would also render the county councils legally responsible for the establishment and maintenance of the dispensaries, receiving from the Insurance Commissioners payments in respect of insured persons admitted to treatment there, they recognise the value of the many voluntary private agencies which are already partly covering the field of endeavour. They accordingly suggest that approved societies and charities of this kind might be represented on consultative committees to be established for the guidance of the county councils. This suggestion strikes us as a very weak spot in an otherwise excellent Report; for it would probably lead to a *maximum* of friction with a *minimum* of practical result. Still, the mere fact that voluntary agencies are not to be snubbed out of existence altogether by officialdom is something to go on with; and some more sensible proposal for utilising them profitably will be expected when the final Report of the Tuberculosis Committee appears.

